# MFG-E8-Derived Oligopeptide MOP3 Facilitates Anti-Inflammatory M2-like Macrophage Polarization in Gut Ischemia/Reperfusion

**DOI:** 10.3390/cells15070606

**Published:** 2026-03-29

**Authors:** Russell Hollis, Yuichi Akama, Yongchan Lee, Jingsong Li, Megan Tenet, Monowar Aziz, Ping Wang

**Affiliations:** 1 Center for Immunology and Inflammation, The Feinstein Institutes for Medical Research, Manhasset, NY 11030, USA; 2 Department of Surgery, Zucker School of Medicine, Manhasset, NY 11030, USA; 3 Elmezzi Graduate School of Molecular Medicine, Northwell Health, Manhasset, NY 11030, USA

**Keywords:** M1, M2, macrophage, MFG-E8, MOP3, gut ischemia/reperfusion

## Abstract

**Highlights:**

**What are the main findings?**

- MOP3 treatment in gut ischemia/reperfusion injury increases M2-like macrophage polarization.
- Clearance of eCIRP by macrophages via MOP3/α_v_β_3_ drives M2-like polarization.

**What are the implications of the main findings?**

- Macrophage polarization represents a new downstream mechanism of the therapeutic peptide MOP3.
- MOP3 may have applications in other intestinal diseases through this.

**Abstract:**

Gut ischemia/reperfusion (I/R) injury releases damage-associated molecular patterns (DAMPs), such as extracellular cold-inducible RNA-binding protein (eCIRP). Milk fat globule–epidermal growth factor VIII-derived oligopeptide 3 (MOP3) is a novel peptide enabling macrophage uptake of eCIRP via αvβ3-integrin. MOP3 reduces inflammation in gut I/R, but its mechanisms are not completely understood. We hypothesized MOP3 promotes macrophage polarization toward an anti-inflammatory, M2-like phenotype in gut I/R. We induced gut I/R in mice through 60 min of superior mesenteric artery occlusion followed by 4 h of reperfusion. Intestines were evaluated for macrophage polarization by flow cytometry and immunofluorescence histology. Peritoneal cavity macrophages were isolated from mice and treated with eCIRP, MOP3, α_v_β_3_-antibody, and/or naïve IgG for 4 or 24 h. Polarity was assessed by flow cytometry, qPCR, and ELISA. Compared to the sham, the M2 proportion after gut I/R decreased by 22.7%, and the M1 proportion increased by 241%. MOP3 treatment increased the M2 proportion by 64.3%, and the M1 proportion decreased by 22.7%. In eCIRP-stimulated macrophages, MOP3 treatment increased M2-like and reduced M1-like cell-surface markers, gene expression, and cytokine levels. α_v_β_3_ antibody dramatically reduced MOP3′s effects. MOP3 promotes M2 polarization through α_v_β_3_ integrin-mediated clearance of eCIRP, a novel mechanism whereby MOP3 reduces gut I/R injury.

## 1. Introduction

Ischemia/reperfusion (I/R) injury occurs when an organ is deprived of blood supply (ischemia) and subsequently experiences rapid restoration of blood flow (reperfusion) [1]. Reperfusion allows for systemic circulation of damage-associated molecular patterns (DAMPs) and other distress signals, which are released as a result of cell death from tissue ischemia [1,2]. DAMPs initiate inflammatory pathways through receptor–ligand interactions, causing pathological inflammation with the potential for organ injury, critical illness, and death [3]. Gut I/R, a severe form of I/R, develops from acute occlusion of the superior mesenteric artery (SMA), which affects 1 in every 1000 hospitalized patients [4]. Patients with prothrombotic conditions, such as tobacco use, and patients prone to embolic events, such as those with atrial fibrillation, are at highest risk [5]. I/R injury can also occur after a strangulated segment of the small intestine is surgically or manually reduced from an incarcerated hernia of the abdominal wall [6,7]. Treatment of gut ischemia requires restoration of perfusion and often requires surgical or endovascular techniques to relieve any vascular obstruction [4,6]. To date, there are no FDA-approved therapies that specifically attenuate reperfusion injury, which relegates this stage of treatment to supportive care, often in the intensive care unit.

A key DAMP in gut I/R is extracellular cold-inducible RNA-binding protein (eCIRP), which activates toll-like receptor 4 (TLR4) and triggering receptor expressed on myeloid cells-1 (TREM-1) to upregulate cytokine expression and release [8,9]. Consequently, neutralization of eCIRP has gained interest as a therapeutic strategy to reduce I/R injury [10,11]. Treatments that prevent eCIRP/receptor interactions through competitive inhibition, noncompetitive inhibition, or binding to eCIRP have shown efficacy in this disease [11]. More recently, milk fat globule–epidermal growth factor VIII-derived oligopeptide 3 (MOP3), a novel small peptide that facilitates clearance of eCIRP by macrophages, has emerged [10,12].

Macrophages are key factors in gut homeostasis and disease [13]. Resident macrophages in the intestine participate in several regulatory activities, such as clearance of apoptotic cells, maintenance of the mucosal barrier, and production of cytokine signals [13]. The balance of cytokines produced by macrophages, including tumor necrosis alpha (TNFα) and interleukin-10 (IL-10), affects the degree of inflammation or healing in the intestine [13,14]. Therefore, the phenotypic expression and activity of macrophages are both important areas of study in diseases of the gut and potential therapeutic levers to treat gut inflammation. In general terms, activated macrophages are either pro-inflammatory (M1-like) or anti-inflammatory (M2-like) [15]. M1-like macrophages are primarily responsible for upregulating inflammatory cytokines, such as TNFα and IL-6, and are characterized by markers, such as CD86, and enzymes, such as inducible nitric oxide synthase (iNOS) [15,16]. Conversely, M2-like macrophages upregulate cytokines responsible for healing, such as IL-10, and are characterized by markers, such as CD163, and enzymes, such as arginase-1 (Arg-1) [15,16].

The relative proportions of M1-like and M2-like macrophage populations can fluctuate with inflammatory insults such as I/R injury, leading to a pathological shift toward excessive inflammation. This progression toward an M1-predominant milieu exacerbates tissue damage and is initiated by various stimuli, including lipopolysaccharide (LPS), interferon gamma (IFN-γ), and TNFα [17]. However, polarization of macrophages toward an M2-like phenotype attenuates inflammation and initiates healing [18]. Thus, treatments that reduce injury during I/R may do so in part by shifting the macrophage population to an M2-predominant phenotype. Previously, MOP3 has demonstrated efficacy in attenuating inflammation using a mouse model of gut I/R [10]. The MFG-E8-derived peptide employs the phagocytic properties of macrophages by linking eCIRP with α_v_β_3_ integrin, promoting clearance of the DAMP [10]. Previous studies have evaluated the role of efferocytosis on polarization, which is, in part, facilitated by MFG-E8 [19,20,21]. Efferocytosis, they reported, may initiate a cascade of intracellular events that promotes M2 polarization [19,20,21]. However, the effects of this process on the macrophage phenotype and regulation of intestinal healing and homeostasis are unknown.

Given our current understanding of the peptide, we hypothesized that MOP3-facilitated phagocytosis of eCIRP polarizes macrophages toward an M2-like phenotype in gut I/R to suppress inflammation and promote healing. To test our hypothesis, we first evaluated the intestinal macrophage population for M1 and M2 polarization after gut I/R and treatment with either vehicle or MOP3. After establishing MOP3′s effect on polarization in vivo, we treated peritoneal macrophages with eCIRP and MOP3 to determine if engulfment of eCIRP via MOP3 was responsible for the increased percentage of M2-like macrophages relative to M1. Additionally, we measured gene expression and cytokine production after eCIRP and MOP3 treatment. Lastly, we inhibited MOP3-α_v_β_3_-integrin interactions to determine the effect of this interaction on pro- and anti-inflammatory cytokine production. Overall, our findings suggest that MOP3 treatment affects macrophage phenotype and activity via M2 polarization, which we observed in cell cultures and our translational gut I/R model.

## 2. Materials and Methods

### 2.1. Protein and Peptide Synthesis and Preparation

MOP3 (RGDSSSYKTWNLRAFGWY) was designed by us and synthesized by Genscript (Piscataway, NJ, USA) to a purity ≥95% and stored in a lyophilized form at −20 °C [12]. For use in experiments, a stock tube of 1 mg of lyophilized MOP3 was dissolved in 100 µL of DMSO for stock solution, which was then diluted in a 4:1 (*v*:*v*) ratio with PBS (in vitro) or normal saline (NS; in vivo) to create a working solution with a final concentration of 2 mg/mL. eCIRP was synthesized in our lab as previously described or purchased commercially from Cusabio Technology, LLC (Houston, TX, USA; Code CSB-EP005440MO) [8].

### 2.2. Experimental Animals

C57BL/6 male mice aged 8–13 weeks (wk) (Charles River Laboratories, Wilmington, MA, USA) were housed in a standard laboratory environment with a 12 hour (h) alternating light/dark cycle. They were fed a standard mouse-chow diet and allowed a minimum of 3 days to acclimate before experiments. All animal experiments were approved by the Institutional Animal Care and Use Committee (IACUC) at the Feinstein Institutes for Medical Research.

### 2.3. Gut Ischemia/Reperfusion Injury Model

Mice underwent gut I/R as previously described [10]. Briefly, mice were anesthetized with 2–4% inhaled isoflurane and evaluated for absent pedal reflex and decreased respiratory rate. A midline incision was used to expose the peritoneal cavity, after which the small and large intestines were eviscerated and retracted to identify the SMA, which was occluded with an atraumatic vascular clip (Item No. 18055-04; Fine Science Tools, Foster City, CA, USA). The abdomen was covered with a warm saline-soaked gauze for 60 min (min), after which the clip was removed, the incision was closed, and 200 µL MOP3 (20 mg/kg) or vehicle (DMSO dissolved in NS at the same ratio as the treatment) was delivered by retro orbital injection. This dose of MOP3 was previously established as an effective dose in sepsis, necrotizing enterocolitis, and gut and hepatic I/R models [10,11,22]. At that single optimized dose, we observed statistically significant attenuation of gut I/R and induction of M2 macrophage polarization. Consequently, we established this as the optimized dose for our study. Buprenorphine (0.05 mg/kg) and normal saline (0.5 mL) were injected subcutaneously before the mice were returned to their cages for recovery. A circulating water heater was used throughout surgery and while the mice recovered. Mice were provided with hydrogel and diet cups for 4 h before they were sacrificed for specimen collection.

### 2.4. Cell Culture and Treatment

For in vitro experiments, peritoneal cavity macrophages were collected from mice after the mice were euthanized by carbon dioxide inhalation. Phosphate-buffered saline (PBS) with 2% fetal bovine serum (FBS) and 1% penicillin streptomycin (Pen/Strep) by volume were instilled into the peritoneum of mice two times, 10 mL at a time, using an 18-gauge needle. This lavage fluid was centrifuged for 10 min at 300× *g* and 4 °C to pellet the cells. Samples with visible red blood cells (RBCs) were treated with RBC lysis buffer (Cat. 555899; BD Biosciences, Franklin Lakes, NJ, USA) for 2 min before addition of Roswell Park Memorial Institute (RPMI) media. Cells were then seeded with RPMI supplemented with 10% FBS, 1% Pen/Strep, 2% l-glutamine, and 25 millimolar (mM) HEPES buffer. After 2–3 h, cells were washed with warm PBS to isolate macrophages via the adhesion method. Supplemented RPMI medium was replaced for overnight incubation before treatment.

Depending on the experiment, cells were treated with eCIRP (1 µg/mL) and/or MOP3 (10 µg/mL) for 4 or 24 h. In vitro dosing was determined by previously published dose–response experiments using RAW 264.7 cells, which show 10 µg/mL as an effective therapeutic dose [12]. PBS was used as a control group. Treatment-supplemented medium was incubated for 30 min at room temperature before being administered to the cells. For cytokine detection experiments, separate groups of eCIRP and MOP3-treated cells were pretreated with IgG (10 µg/mL; bs-0295p; Bioss, Woburn, MA, USA) or α_v_β_3_ polyclonal ab (10 µg/mL; cat. bs-1310r; Bioss). In flow cytometry experiments, eCIRP was labeled with fluorescein isothiocyanate (FITC) using the FITC Conjugation Kit (Fast)—Lightning-Link (Cat. ab188285, Abcam, Cambridge, UK).

### 2.5. Flow Cytometry

Peritoneal macrophages (10^5^ cells/well) were collected with a cell scraper after washing with PBS. Cells were placed into 5 mL tubes and incubated in TruStain FcX PLUS Antibody (Cat. 156604; BioLegend, San Diego, CA, USA) at a concentration of 0.5 µL/100 µL for 20 min at 4 °C. Cells were then washed with fluorescence-activated cell-sorting (FACS) buffer, centrifuged for 10 min at 300× *g* 4 °C, and stained with the following fluorophore-conjugated antibodies for 30 min: F4/80-APC (Cat. 123116; BioLegend), CD86-BV421 (Cat. 105032; BioLegend), and CD163-PerCP-eFluor710 (Cat. 46-1631-82; Thermo Fisher Scientific, Waltham, MA, USA). After incubation, the cells were washed again with FACS buffer, centrifuged for 10 min at 300× *g* 4 °C, and resuspended in fixation buffer (4% paraformaldehyde) for analysis.

Intestinal macrophages from sham mice and mice who underwent gut I/R were resuspended in PBS in 5 mL tubes after isolation, followed by centrifugation for 10 min at 300× *g* 4 °C. To reduce nonspecific-binding and detect live cells, samples were resuspended in PBS with TruStain FcX PLUS Antibody and Zombie Aqua Fixable Viability Kit (Cat. 423101; BioLegend) at concentrations of 0.5 µL/100 µL for 20 min at 4 °C. The cells were then washed with PBS and centrifuged for 10 min at 300× *g* 4 °C before staining with F4/80-APC, CD86-BV421, and CD163-PerCP-eFluor710. After all staining steps, samples were resuspended in fixation buffer for analysis. For both experiments, macrophages were identified by F4/80 positivity—M1 by CD86 positivity and M2 by CD163 positivity.

**Figure A1:**
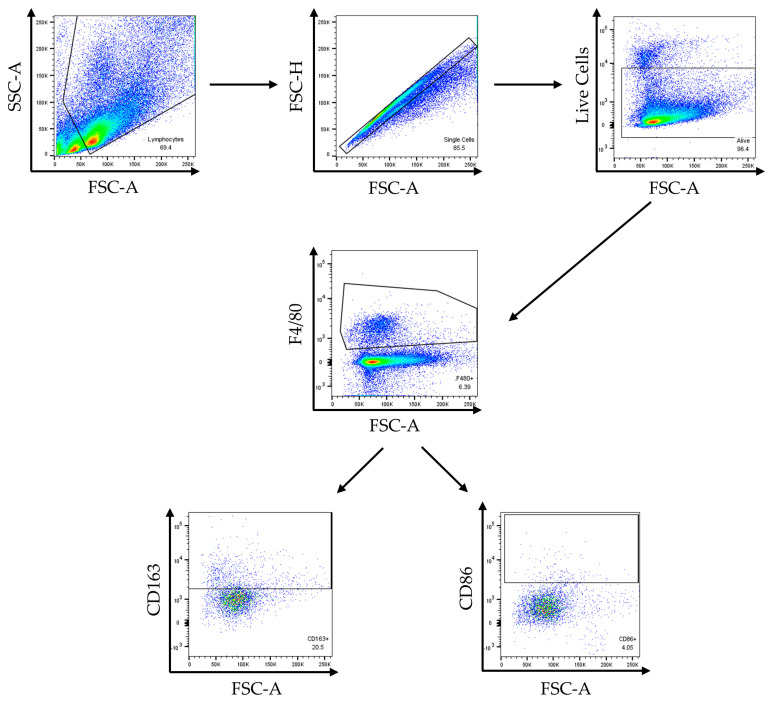
Gating hierarchy for experiments in Figure 1. This sample is from a mouse in the sham group of the gut I/R experiment and reflects the gating strategy to identify M2-like and M1-like macrophages. Gates represent the cells selected for the subsequent scatter plot and/or positivity for the labeled marker of interest.

**Figure A2:**
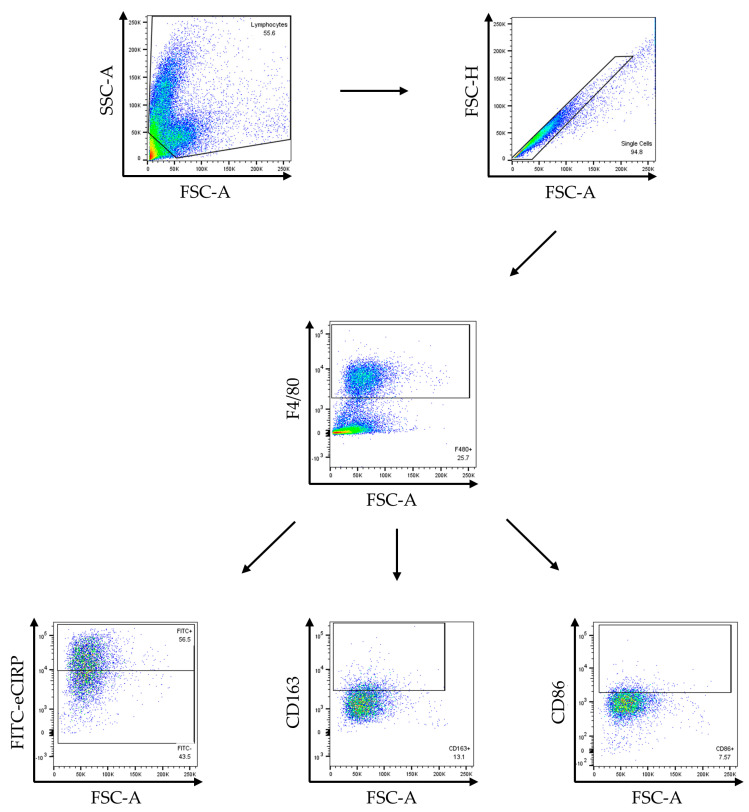
Gating hierarchy for experiments in Figure 3. This sample is from peritoneal macrophages treated with eCIRP and MOP3, reflecting the gating strategy to identify FITC-eCIRP-positive macrophages, M2-like macrophages, and M1-like macrophages. Gates represent the cells selected for the subsequent scatter plot and/or positivity for the labeled marker of interest.

The gating strategy for flow cytometry experiments included exclusion of doublets by gating forward scatter height (FSC-H) by forward scatter area (FSC-A) and removing observations with a high area-to-height ratio from our analysis. Fluorescence-minus-one (FMO) controls were used to determine the threshold of positivity for M1 and M2 markers. Compensation of fluorophores was performed using single-stained compensation beads for each fluorophore. Full gating hierarchies are available in Appendix A Figure A1 (in vivo) and Figure A2 (in vitro). Data were acquired with BD Symphony flow cytometer and analyzed with FlowJo_v10.10.0 software (Tree Star, Ashland, OR, USA).

### 2.6. Immunofluorescence Histology

Intestinal specimens were collected after gut I/R and stored in 10% formalin. Slides with intestinal tissue were prepared by AML Laboratories (St. Augustine, FL) in 5-micron sections. Samples were deparaffinized with xylene and rehydrated in ethanol. Antigen retrieval was performed with heated citric acid buffer (Cat. H-3300; Vector Labs; Newark, CA, USA). The samples were then washed in TBS and 0.1% triton x-100 (TX 100). After circumscribing the samples with an ImmEdge pen (cat. H-4000; Vector Lab), blocking was performed with 3% bovine serum albumin in TBS and 0.02% TX 100 for 1 h at room temperature. Samples were stained in fluorophore-conjugated primary antibodies for 24 h in 4 °C: F4/80-APC, CD86-PE (cat. 105105; Biolegend), and CD163-PerCP-eFluor710. Samples were washed again with TBS and 0.02% TX 100 three times before counterstaining nuclei with NucBlue Hoechst 33342 (Invitrogen Life Technologies, Eugene, OR, USA). Samples were washed again before mounting with a cover glass and Prolong gold antifade (Thermo Fisher Scientific, Waltham, MA, USA). Images were captured at 630× magnification with a Nikon Eclipse Ti-S microscope equipped with an LSM900 confocal unit (Nikon Instruments, Melville, NY, USA).

### 2.7. Real-Time Quantitative Polymerase Chain Reaction (RT-qPCR)

RNA was isolated from peritoneal macrophages 24 h after treatment using a Cytiva RNAspin Mini Kit (Cat. 25050072, Marlborough, MA, USA) according to the manufacturer’s protocol using two wells of 5 × 10^5^ macrophages for a total of 10^6^ cells per sample, eluted in 20 µL of diethyl pyrocarbonate (DEPC) water. A total of 0.5 µg of RNA per sample was used for reverse transcription. RT-qPCR was performed with QuantStudio 3 (Applied Biosystems, Waltham, MA, USA). The following genes with forward and reverse primer sequences were used: β-Actin 5′-CGTGAAAAGATGACCCAGATCA-3′ and 5′-TGGTACGACCAGAGGCATACAG-3′, Arginase-1 5′-ATTATCGGAGCGCCTT-3′ and 5′-CGTGGTCTCTCACGTCATACT-3′, iNOS 5′-GGCAAACCCAAGGTCTACGTT-3′ and 5′-GAGCACGCTGAGTACCTCATTG-3′.

### 2.8. Enzyme-Linked Immunosorbent Assay (ELISA)

IL-10, IL-6, and TNFα assays were performed according to the manufacturer’s protocol using overnight sample incubation at 4 °C. Supernatants were collected from a 96-well plate with 10^5^ peritoneal macrophages per well treated for 24 h.

### 2.9. Statistical Analysis

Results from each experiment were analyzed with GraphPad Prism https://www.graphpad.com/ (San Diego, CA, USA). Data were tested for normality or log normality by Shapiro–Wilk test and QQ plot analysis. Data that passed normality tests are represented by mean ± SEM. Comparisons were performed using an unpaired *t*-test, ordinary one-way analysis of variance (ANOVA), and Tukey’s multiple-comparison test where applicable. Data that did not pass normality tests are represented by median ± IQ range and were tested by Kruskal–Wallis test and Dunn’s multiple-comparison test. *p*-values < 0.05 were considered statistically significant.

## 3. Results

### 3.1. Gut I/R Promotes M1 over M2 Polarization, Which Is Reversed by MOP3

**Figure 1:**
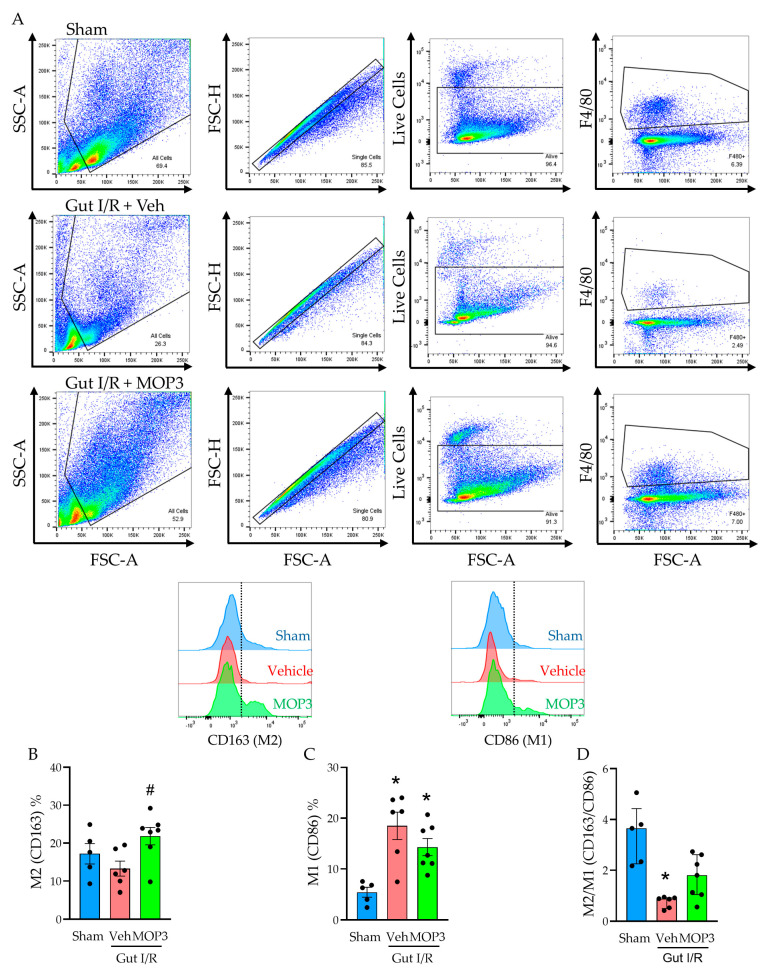
MOP3 increases the M2/M1 ratio of intestinal macrophages in gut I/R. (**A**) Representative gating is shown for live macrophages from the small intestines of sham mice and mice that underwent gut I/R. Boxes on dot plots indicate the cell populations of interest, and the dashed lines on the histograms indicate the threshold for positivity of the selected marker. (**B**–**D**) Flow cytometry analysis is shown for sham-, vehicle-, and MOP3-treated mice after gut I/R represented by (**B**) percentage of M2 (CD163^+^) cells per group, (**C**) percentage of M1 (CD86^+^) cells per group, and (**D**) the M2/M1 (%CD163/%CD86) ratio. Results from each group were evaluated for normality by Shapiro–Wilk test and QQ plot. Results that passed normality (**B**,**C**) are expressed as mean ± SEM and were evaluated by ordinary one-way ANOVA and Tukey’s multiple-comparisons test (* *p* < 0.05 vs. Sham, ^#^ *p* < 0.05 vs. Veh). Results that did not pass normality (**D**) are expressed as median ± IQ range and were evaluated by Kruskal–Wallis ANOVA and compared with Dunn’s multiple-comparison test (* *p* < 0.05 vs. Sham).

To determine the effect of gut I/R on macrophage polarization, we compared the percentages of M2-like (F4/80^+^CD163^+^) and M1-like (F4/80^+^CD86^+^) macrophages in the small intestines of mice after sham surgery or gut I/R using flow cytometry (Figure 1A). Compared to sham mice, the M2 proportion in gut I/R mice decreased by 22.7% (Figure 1B), whereas the M1 proportion increased by 241% (Figure 1C; *p* < 0.01), reflecting the acute inflammatory phase of I/R injury. However, with MOP3 treatment after gut I/R, the M2 proportion increased by 64.3% (Figure 1B; *p* < 0.05), and the M1 proportion decreased by 22.7% (Figure 1C), demonstrating an abrogated inflammatory response and transition toward healing. Taking these results into account, we calculated the ratio of M2-like to M1-like macrophages in each group to further assess the balance between inflammation and healing. Compared to the sham, the M2/M1 ratio decreased by 77.6% after gut I/R (*p* < 0.01) but then increased by 127% with MOP3 treatment compared to vehicle (Figure 1D). These data show that gut I/R induces an inflammatory phenotype in macrophages, whereas MOP3 attenuates this reaction and stimulates macrophages to adopt an anti-inflammatory phenotype.

**Figure 2:**
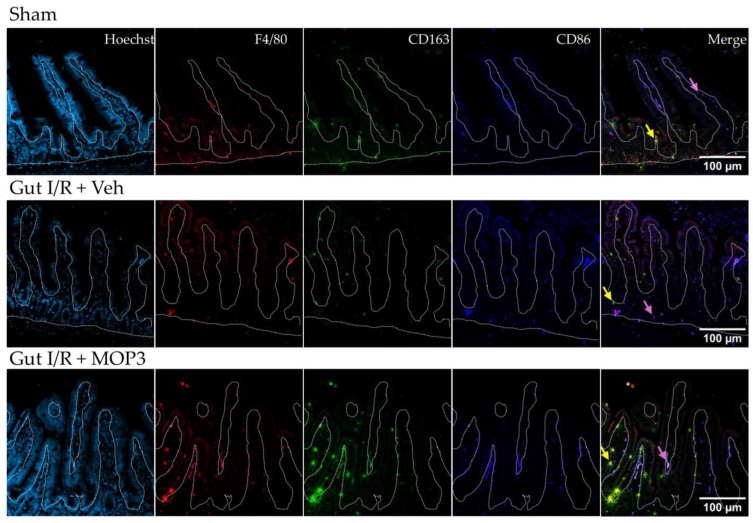
MOP3 promotes M2 macrophage over M1 macrophage populations in the intestinal lamina propria after gut I/R injury. Representative images of intestines from sham mice or mice who underwent gut I/R with vehicle or MOP3 treatment are shown. Immunofluorescence was performed with fluorophore-conjugated antibodies for F4/80 (macrophage, red), CD163 (M2, green), and CD86 (M1, dark blue). Hoechst (light blue) shows nuclear counterstaining. In the merged slides, yellow indicates F4/80^+^CD163^+^ cells (M2 macrophages, yellow arrow), and purple indicates F4/80^+^CD86^+^ cells (M1 macrophages, purple arrow). The white dotted line indicates the lamina propria layer of the small intestines.

To support these findings, we qualitatively assessed the small-intestine histology by immunofluorescence. Here, again, we observed a predominant M2 population and low M1 population in the intestines of sham mice (Figure 2). However, after gut I/R, the M1 population increased relative to the M2 population (Figure 2). Lastly, MOP3 treatment demonstrated an increase in M2-like macrophages over M1-like macrophages. Notably, we observed these changes within the lamina propria layer of the small intestines where macrophages are most abundant [13]. Correlating with our flow cytometry findings, these data further demonstrate the shift in macrophage phenotype experienced in gut I/R toward M1 and the converse shift to M2 with MOP3 treatment.

### 3.2. MOP3-Facilitated Uptake of eCIRP Promotes M2 over M1 Polarization

**Figure 3:**
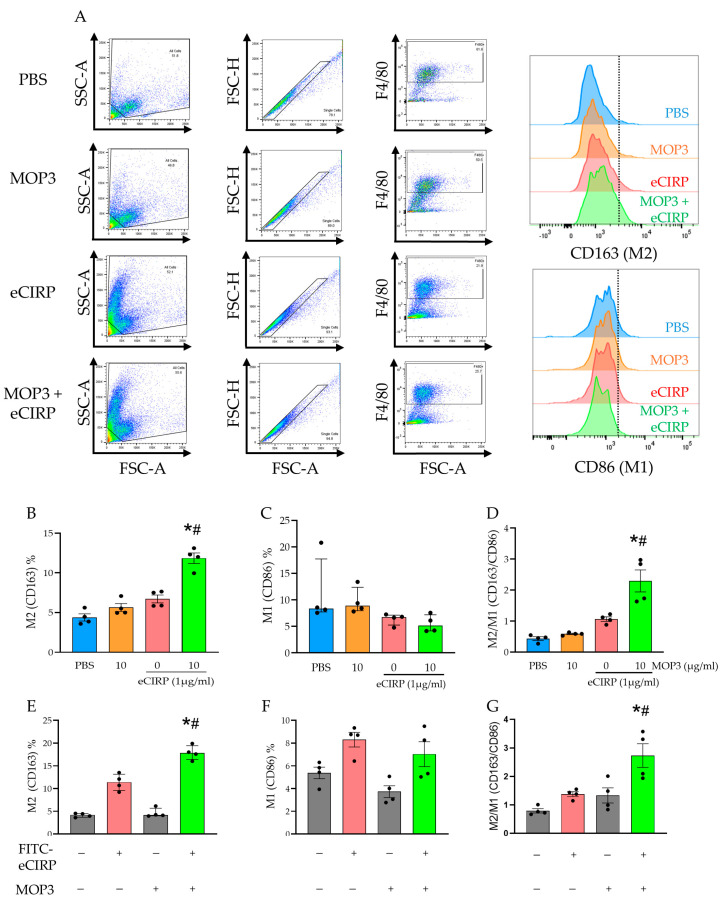
MOP3-facilitated uptake of eCIRP increases the M2/M1 macrophage ratio. (**A**) The gating strategy for flow cytometry of peritoneal macrophages treated with FITC-eCIRP and/or MOP3 is displayed, with M2 represented by CD163 and M1 represented by CD86. Boxes on dot plots indicate the cell populations of interest, and the dashed lines on the histograms indicate the threshold for positivity of the selected marker. (**B**–**D**) Percentage of cells positive for M2 and M1 markers for the respective groups, as well as the M2/M1 ratio, represented by %CD163/%CD86. Results were evaluated for normality by Shapiro–Wilk test and QQ plot. Results that passed normality tests (**B**,**D**) were evaluated by ordinary one-way ANOVA and compared by Tukey’s multiple-comparison test and are expressed as mean ± SEM. Results that did not pass normality tests (**C**) were evaluated by Kruskal–Wallis ANOVA (* *p* < 0.05 vs. PBS, ^#^ *p* < 0.05 vs. 0 (eCIRP)) and are expressed as median ± IQ range. (**E**–**G**) Macrophages in groups treated with FITC-eCIRP and/or MOP3 were classified by FITC-eCIRP positivity/uptake (gray indicates no uptake, red indicates uptake in the group treated with FITC-eCIRP alone, and green indicates uptake in the group treated with FITC-eCIRP and MOP3). Axis labels with −/+ indicate absence/presence of FITC-eCIRP uptake or MOP3 treatment. These groups were assessed for percentage of (**E**) M2 and (**F**) M1 markers, as well as the (**G**) M2/M1 ratio. Results were evaluated for normality by Shapiro–Wilk test and QQ plot. Results that passed normality tests (**F**,**G**) were evaluated by ordinary one-way ANOVA and compared by Tukey’s multiple comparison test and are expressed as mean ± SEM. Results that did not pass normality tests (**E**) are expressed as median ± IQ range and were evaluated by Kruskal–Wallis ANOVA and compared by Dunn’s multiple-comparison test (* *p* < 0.05 vs. FITC-eCIRP^−^MOP3^+^, ^#^ *p* < 0.05 vs. FITC-eCIRP^+^MOP3^−^).

After demonstrating in vivo shifts in M1-like (CD86^+^) and M2-like (CD163^+^) macrophage populations after gut I/R and MOP3 treatment, we assessed MOP3′s effects on peritoneal macrophage phenotypes in vitro (Figure 3A). While the percentage of M2-like macrophages treated with FITC-eCIRP increased by 53.1% compared to PBS-treated macrophages, the M2 percentage in MOP3-treated macrophages increased further by 76.3% compared to FITC-eCIRP alone (Figure 3B; *p* < 0.01). In addition, the median proportion of M1-like macrophages decreased by 23.4% with MOP3 treatment compared to eCIRP alone (Figure 3C). These combined results translated to a 2.1-fold increase in the M2/M1 ratio with MOP3 treatment (*p* < 0.01, Figure 3D). Taking this further, when we compared FITC-positive macrophages (indicating eCIRP clearance by uptake) with FITC-negative macrophages, we observed significantly elevated levels of CD163 among the macrophages that cleared FITC-eCIRP (Figure 3E). Most interestingly, in the MOP3-treated group, we observed a 3.87-fold increase in the median proportion of M2-like macrophages when comparing FITC-eCIRP^+^ to FITC-eCIRP^−^ macrophages, as shown in Figure 3E (*p* < 0.01).

We also observed nonsignificant increases in the CD86^+^ proportion among FITC-eCIRP^+^ macrophages compared to FITC-eCIRP^−^ macrophages in both the untreated and MOP3-treated groups, indicating a lower degree of activation in the FITC-eCIRP^−^ macrophages (Figure 3F). Taking these results together, we again assessed the M2/M1 ratio and observed a 2.1-fold increase after MOP3 treatment compared to eCIRP alone (Figure 3G). In conclusion, these results indicate that MOP3′s function of facilitating macrophage clearance of eCIRP is a key factor in polarization toward an anti-inflammatory phenotype.

### 3.3. MOP3 Reduces M1 Activity While Increasing M2 Activity

**Figure 4:**
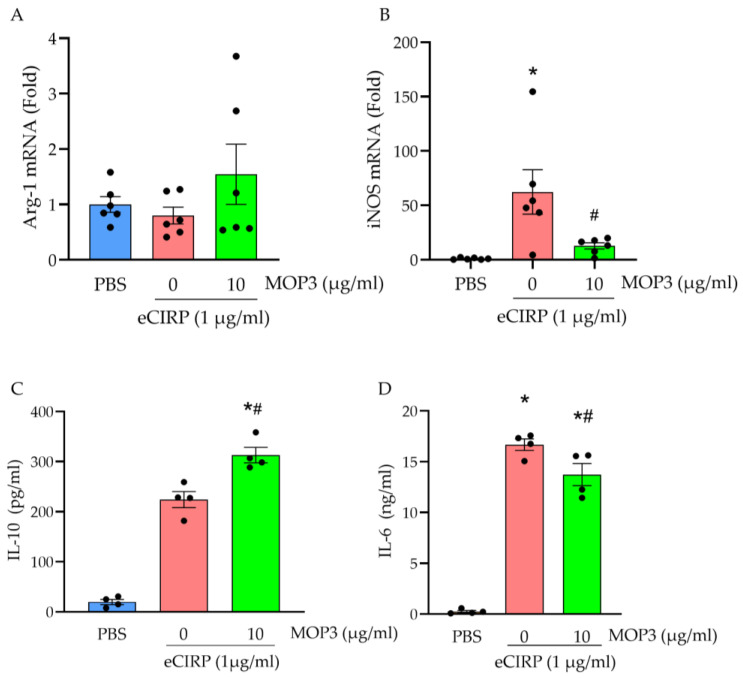
MOP3 alters gene transcription and soluble protein markers in favor of M2-like polarization in eCIRP-stimulated macrophages. Peritoneal macrophage mRNA expression after 24 h of treatment is reported for (**A**) Arg-1 (M2) and (**B**) iNOS (M1). (**C**,**D**) IL-10 and IL-6 concentrations of the supernatant from peritoneal macrophages treated with eCIRP (1 µg/mL) and/or MOP3 (10 µg/mL) were measured by ELISA at 24 h. Data are expressed as mean ± SEM. Results were tested for normality by Shapiro–Wilk test and QQ plot. Results were evaluated by ANOVA and Tukey’s multiple-comparisons test (* *p* < 0.05 vs. PBS, ^#^ *p* < 0.05 vs. 0 (eCIRP)).

In the previous results, we used cell-surface markers to measure both in vivo and in vitro changes to the macrophage phenotype after MOP3 treatment. In the following experiment, we assessed gene expression signatures to differentiate M1 and M2 macrophage populations after eCIRP stimulation and MOP3 treatment. Arginase-1 (Arg-1) expression classically represents the anti-inflammatory M2 phenotype, while inducible nitric oxide synthase (iNOS) more classically represents M1 [23]. In peritoneal macrophages, a 21% decrease in Arg-1 expression was observed from the PBS group to the eCIRP-stimulated group, but MOP3 treatment resulted in a 93% increase in Arg-1 gene expression compared to eCIRP alone (Figure 4A; *p* = 0.287). A more significant change was observed in iNOS, which classically represents M1. To this end, macrophages treated with eCIRP, on average, had a 62.3-fold increase in iNOS gene expression compared to PBS (Figure 4B; *p* < 0.01). When treated with MOP3, there was a 4.9-fold decrease compared to eCIRP alone (Figure 4B; *p* < 0.05).

In addition to gene expression, we also evaluated the production of soluble markers related to M2 and M1 activity. When stimulated with eCIRP, the average concentrations of both IL-10 and IL-6 (Figure 4C,D) in the supernatants of peritoneal macrophages were significantly higher than those of macrophages treated with PBS (*p* < 0.01 and *p* < 0.01, respectively). However, with MOP3 treatment, the concentration of IL-10 in supernatants increased even further by 39.5% compared to eCIRP alone (*p* < 0.01). Conversely, the IL-6 concentration in the supernatants decreased by 17.6% (*p* < 0.05). Taken together, these data indicate that MOP3 amplifies M2 anti-inflammatory activity while attenuating M1 inflammatory activity.

### 3.4. MOP3 Promotes M2 Polarization via Interaction with α_v_β_3_ Integrin

**Figure 5:**
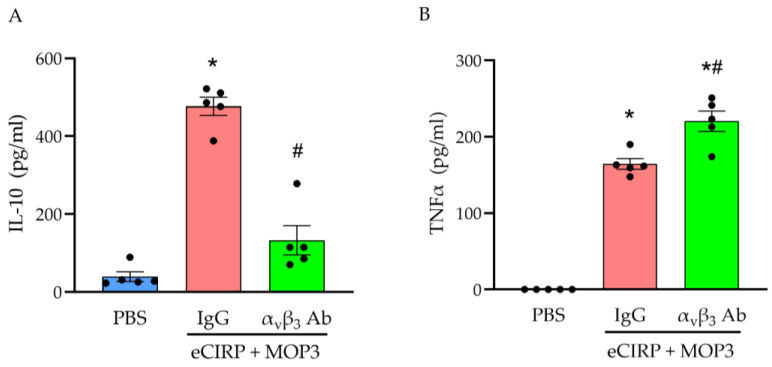
MOP3 increases M2 activity and decreases M1 activity in an α_v_β_3_-dependent manner. Peritoneal macrophages from adult mice were treated with PBS or eCIRP (1 µg/mL) and MOP3 (10 µg/mL). Groups treated with eCIRP and MOP3 were pretreated with either IgG or α_v_β_3_ antibody. Supernatant was collected at 24 h and measured for (**A**) IL-10 and (**B**) TNFα by ELISA. Data are expressed as mean ± SEM. Results were tested for normality by Shapiro–Wilk test and QQ plots. Results were evaluated by ANOVA and Tukey’s multiple-comparisons test (* *p* < 0.05 vs. PBS, ^#^ *p* < 0.05 vs. IgG).

Lastly, once we observed these changes in phenotypic activity after MOP3 treatment, we further explored the mechanism of macrophage polarization by MOP3. To this end, we inhibited the interaction between MOP3 and α_v_β_3_ integrin via antibody blockade. From previous work, this inhibition is known to decrease the uptake of eCIRP by macrophages via MOP3 [1]. Compared to PBS-treated macrophages, macrophages treated with eCIRP, MOP3, and IgG had dramatically higher concentrations of IL-10 in the supernatants—a 12.1-fold increase (Figure 5A; *p* < 0.01). However, when IgG was replaced with α_v_β_3_ antibody, there was a dramatic decrease 72.2% in IL-10 concentration (Figure 5A; *p* < 0.0001). When measuring TNFα, concentrations increased significantly in the supernatants of macrophages treated with eCIRP, MOP3, and IgG compared to PBS (Figure 5B; *p* < 0.01). With α_v_β_3_ antibody treatment, TNFα concentrations increased even further by 34% (Figure 5B; *p* = 0.0018). These data provide evidence that MOP3′s interaction with α_v_β_3_ integrin, which enables uptake of eCIRP by the macrophage, has a key role in the peptide’s ability to promote polarization to M2-like rather than M1-like macrophages.

## 4. Discussion

**Figure 6:**
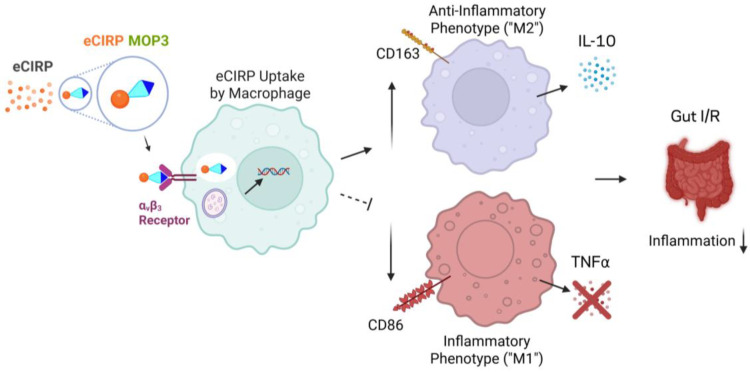
MOP3 clearance of eCIRP promotes M2 polarization of macrophages in mesenteric ischemia/reperfusion injury. MOP3 facilitates phagocytosis of eCIRP by macrophages via α_v_β_3_ integrin, which leads to an increase in polarization toward M2 relative to M1. This phenomenon is demonstrated in mesenteric ischemia/reperfusion, improving healing while decreasing inflammation.

Acute gut I/R causes severe intestinal injury and disruption of homeostasis through sterile inflammation, which can ultimately lead to bacterial translocation and sepsis [4,24]. Macrophages are critical to maintaining gut homeostasis and responding to injury [13]. Whether the response is deleterious or beneficial depends on the activity of the resident macrophages, which is determined by their polarity as pro-inflammatory, i.e., M1-like, or anti-inflammatory, i.e., M2-like [15]. Macrophage polarization influences the trajectory of both acute and chronic intestinal diseases, with M2 anti-inflammatory activity contributing to improved outcomes via mediators IL-10 and TGF-β [18,25,26]. We previously developed a peptide, MOP3, that enables macrophages to clear eCIRP, a DAMP that exacerbates gut I/R and increases M1 polarization [10,27]. In this study, we demonstrated the ability of MOP3 to increase M2 polarization relative to M1, improving the M2/M1 ratio and, thus, the balance of healing over inflammation. We subsequently demonstrated a correlation between MOP3′s mechanism of clearing eCIRP via α_v_β_3_ integrin and the M2-like macrophage phenotype. These findings reveal a novel mechanism through which MOP3 reduces injury and improves outcomes in acute gut I/R (Figure 6).

While gut ischemia can be treated with resuscitation; anticoagulation; and, if necessary, thrombectomy and intestinal resection, management of reperfusion injury remains a challenge for clinicians and scientists [4,28,29]. Several preclinical efforts have targeted disease mediators such as radical oxygen species, electrolyte derangements, and DAMPs [30]. Recently, macrophage polarization has been explored as a therapeutic mechanism in gut I/R [18,26]. We previously demonstrated the impact of eCIRP on gut I/R, as well as the efficacy of eCIRP-neutralizing therapeutics [10]. Here, we describe the novel mechanism of M2 polarization by MOP3 in the context of acute gut I/R, which further illuminates our understanding of the treatment of this devastating disease. At the same time, we must acknowledge that recent literature has challenged the dichotomous nature of macrophage polarization in favor of a more dynamic model [31,32]. Macrophages have demonstrated plasticity in their ability to display both M1 and M2 characteristics simultaneously and at different time points [31,32]. However, a shift towards anti-inflammation, referred to in this study as the M2-like phenotype, can still prove beneficial in disease recovery.

In critical illness such as I/R, eCIRP primarily acts as a ligand to TLR4 and TREM-1 to upregulate inflammation through increased cytokine production, particularly TNFα, IL-1β, and IL-6 [8,9]. Although not previously demonstrated in gut I/R, previous studies have demonstrated eCIRP’s capacity to polarize macrophages toward an M1 phenotype in acute lung injury, exacerbating injury through amplification of the inflammatory immune response [27]. However, macrophages stimulated by MFG-E8 adopt an anti-inflammatory, M2-like phenotype [21,33]. MOP3 is an oligopeptide derived from MFG-E8, combining an eCIRP-avid sequence in the discoidin domain-2 with the RGD motif for integrin signaling [12]. MOP3 increases internalization of eCIRP by macrophages and decreases injury in gut I/R [10,12]. Based on these findings and the properties of MOP3, we sought to evaluate the impact of this therapeutic peptide on macrophage polarization.

In this study, we found that MOP3 polarizes both intestinal and peritoneal macrophages toward an M2-like phenotype and away from M1, which likely contributes to enhanced recovery from gut I/R. We also showed that peritoneal macrophages polarized by MOP3 treatment increased production of IL-10 and gene transcription of Arg-1 while decreasing the production of IL-6 and gene transcription of iNOS at 24 h. This was further demonstrated with the use of α_v_β_3_ antibody, where inhibition of the MOP3-α_v_β_3_-integrin phagocytosis mechanism resulted in lower production of IL-10 and higher production of TNFα. We chose to inhibit MOP3′s function with an α_v_β_3_ antibody because a previous study from our lab demonstrated α_v_β_3_ integrin as the key mediator for MOP3-induced clearance of eCIRP by macrophages [12]. Regulation of cytokines is important due to their association with further macrophage polarization and activity, with IL-10 upregulating M2 and TNFα upregulating M1 [34]. This pattern of cytokine expression has been observed in other recent studies evaluating the impact of M2 polarization on gut I/R, with researchers finding that M2 polarization is associated with decreased injury and improved outcomes [18,26]. Along with our own findings, this evidence supports the M2 polarization mechanism as a contributor to the beneficial effects observed with MOP3 treatment in gut I/R.

In our mouse model, we chose 60 min of ischemia and 4 h of reperfusion based on our lab’s previous work demonstrating sufficient injury without excessive mortality, minimizing survivor bias [10]. We delivered a one-time dose of MOP3 via retro-orbital injection based on the efficacy of this protocol in previous studies [10,12]. Macrophage polarization can happen at varying time points, with evidence suggesting M2 and M1 phenotypes can appear at both shorter and longer time points, depending on the stimulus [18,27]. Thus, for our in vitro studies, we chose a longer time point of 24 h to confirm the sustained effects of MOP3 on macrophage polarization. In addition, our in vitro studies evaluated the responses of peritoneal macrophages, which have different characteristics than intestinal macrophages, although both respond to systemic and local inflammation from reperfusion injury.

Although we provide evidence of MOP3′s ability to promote M2 polarization in gut I/R, the mechanism driving this process, and the resulting impact on macrophage activity, this study is not without limitations. For example, we did not evaluate all the reported M2 and M1 markers, such as CD80 in M1 and CD206 in M2. However, we found strong evidence that CD163 expression increases, and CD163 is a prominent M2 marker associated with healing, particularly in a subset of M2 macrophages labeled M2c [35]. MOP3 treatment-driven M2 polarization could predominantly be to this subset, i.e., M2c, which produces IL-10 and participates in wound healing. M2c is also the most likely subset of macrophages affected by MOP3 due to its association with increased phagocytic activity [15]. Even though several surface and intracellular markers are widely used for M1 and M2 macrophage detection, our strategy of using select markers, although it does detect macrophage polarization, has the limitation of not including additional markers in this study. Furthermore, there is some controversy about the role of CD163^+^ macrophages in disease. Some sources cite an accumulation of CD163^+^ macrophages in inflammatory bowel disease [36]. However, CD163^+^ macrophages have also demonstrated the ability to phagocytose bacteria and are associated with anti-inflammatory states [37,38]. The different findings reported in different pathologies emphasize the need to evaluate macrophage polarization in disease-specific contexts.

In addition, we did not determine the downstream, intracellular pathway directly responsible for M2-like polarization after phagocytosis. However, previous literature has associated efferocytosis directly with M2 reprogramming, such as via MOP3′s parent protein, MFG-E8 [21,39]. Our data also shows that MOP3 with eCIRP results in higher M2 polarization rates than either MOP3 or eCIRP alone. Lastly, we only used male mice due to sex differences in immune response, especially in the context of gut I/R [40]. While this eliminates confounding effects between the sexes, it also limits the generalizability of our findings.

## 5. Conclusions

This study was the first to evaluate how the clearance of eCIRP impacts macrophage polarity, both in phenotype and function, and how MOP3 drives this process in gut I/R. With these findings, we uncover a new mechanism to explain MOP3′s beneficial effects in gut I/R and thereby show the potential for MOP3 in other diseases that are influenced by the inflammatory or anti-inflammatory state of macrophage populations. Future studies should evaluate MOP3′s impact on M2 populations in other diseases, in female mice, and in larger animal models.

## Data Availability

All data supporting the findings of this study are presented within the manuscript. No additional publicly archived datasets were generated or analyzed during the current study. The corresponding authors, Monowar Aziz and Ping Wang, will gladly provide further information or raw data upon reasonable request and with appropriate justification.

## Abbreviations

The following abbreviations are used in this manuscript:

Arg-1	Arginase-1
DAMP	Damage-associated molecular pattern
DEPC	Diethyl pyrocarbonate
eCIRP	Extracellular cold-inducible RNA-binding protein
ELISA	Enzyme-linked immunosorbent assay
FACS	Fluorescence-activated cell sorting
FBS	Fetal bovine serum
FITC	Fluorescein isothiocyanate
FSC-A	Forward scatter area
FSC-H	Forward scatter height
Pen/Strep	Penicillin streptomycin
I/R	Ischemia/reperfusion
IACUC	Institutional Animal Care and Use Committee
IFN-γ	Interferon gamma
IL-10	Interleukin-10
iNOS	Inducible nitric oxide synthase
LPS	Lipopolysaccharide
Min	Minute
mM	Millimolar
MOP3	Milk fat globule-epidermal growth factor-VIII-derived oligopeptide 3
NS	Normal saline
PBS	Phosphate-buffered saline
RBS	Red blood cells
RPMI	Roswell Park Memorial Institute
RNA	Ribonucleic acid
RT- qPCR	Quantitative polymerase chain reaction
TBS	Tris-buffered saline
TLR4	Toll-like receptor 4
TNFα	Tumor necrosis factor alpha
TREM-1	Triggering receptor expressed on myeloid cells 1
TX 100	Triton x-100
